# School Holiday Food Provision in the UK: A Qualitative Investigation of Needs, Benefits, and Potential for Development

**DOI:** 10.3389/fpubh.2016.00172

**Published:** 2016-08-22

**Authors:** Pamela Louise Graham, Eilish Crilley, Paul B. Stretesky, Michael A. Long, Katie Jane Palmer, Eileen Steinbock, Margaret Anne Defeyter

**Affiliations:** ^1^Department of Psychology, Northumbria University, Newcastle upon Tyne, UK; ^2^Social Sciences and Languages, Northumbria University, Newcastle upon Tyne, UK; ^3^Food Cardiff, Cardiff and Vale of Glamorgan Local Public Health Team, Whitchurch Hospital, Cardiff, UK; ^4^Brakes Group, Ashford, Kent, UK

**Keywords:** food insecurity, children, families, school holidays, holiday clubs

## Abstract

Access to an adequate supply of nutritious food has been recognized as a basic human right. However, many families across the UK face food insecurity, which is thought to be exacerbated during school holidays. To address this issue, some schools and community groups have chosen to roll out holiday clubs, though research into the effectiveness of such interventions is limited and no studies to date have evaluated holiday clubs being organized through schools. In an effort to address some of the limitations in the research literature, the current qualitative investigation utilized semi-structured interviews with staff involved in holiday clubs in school *and* community venues with the aim of gaging their views on the need for and benefits of holiday food provision in addition to potential areas for development. The investigation revealed that staff perceived many families to be facing food insecurity and isolation during the school holidays, which may be alleviated through holiday club provision. Holiday clubs were viewed as a valuable source of support for children and adults, providing food, activities, and learning experiences. Staff were keen to see them implemented on a wider scale in future but suggested some areas that require attention in any future development of such provision. Findings are discussed in relation to current research, policy, and practice surrounding the health and wellbeing of children and families.

## Introduction

Sufficient access to food has long been recognized as a basic human right, which is critical to adequate health and wellbeing, and central to human dignity (1, 2). However, the increasing numbers of people in the UK obtaining food through food banks, where food is provided free of charge to those in crisis, suggests that many people are struggling to meet their basic need for food without additional support. Recent statistics released by the UK food bank network, The Trussell Trust,^1^ showed that 1,084,640 people were given a 3-day emergency food parcel between April 2014 and March 2015. This is more than three times the number of people given food parcels across 2012–2013. Moreover, 37% of those provided with food through The Trussel Trust in 2014–15 were children.

A lack of food security [i.e. “sustained access by all consumers to sufficient food that is affordable, safe, nutritious, and appropriate for an active and healthy life” (3) p. 403] among households with children is concerning given that research has demonstrated a link between poor dietary habits and detrimental health and educational outcomes for children. For example, food insecurity and inferior dietary intake has been associated with poorer academic performance, weight gain (4, 5), and a higher prevalence of dental caries (6). Moreover, poor nutritional habits in childhood have been linked to poorer health outcomes in adulthood (7), thus demonstrating the importance of encouraging children to eat well from a young age.

The importance of adequate food intake for children is evident across UK school food policy. Throughout the school day, children are provided with a range of meals and snacks, including breakfast, lunch, fruit, and milk, all of which must adhere to comprehensive nutritional standards (8). However, school food is generally only accessible for around 39 weeks of the year as UK schools are not under any obligation to provide food for children during the school holidays. This has led to growing concern about the potential for children to suffer “holiday hunger” (9), which refers to the tendency for children to be unable to access an adequate supply of nutritious food during the school holidays.

The potential prevalence of holiday hunger was demonstrated through a recent survey, commissioned by Kellogg’s (10), which found that 39% (*N* = 459) of teachers surveyed in the UK believed that their pupils do not get enough to eat during the school holidays. Furthermore, research drawing on the views of parents has shown that the school holidays are a time of considerable financial difficulty for families, with some parents relying on sporadic meal patterns and cheap foods of low nutritional value to ensure that their children are fed (11, 12).

A lack of engagement in physical activity during the school holidays is another issue that has received recent attention in the UK, with organizations calling for investment in more physical activity provision during the school holidays (13). Participation in physical activity is important as it has been linked to various, favorable health outcomes, including weight loss, blood pressure reduction, and alleviation of depressive symptoms (14). However, previous research has shown that children become more sedentary during the school holidays, which puts them at risk of significant weight gain. A recent UK study (15) involving 463 children aged 8–9 years showed that their levels of cardiorespiratory fitness increased throughout the school term but decreased significantly during the school summer holidays. In the same study, children’s body mass index was shown to remain stable across the school year but increased during the summer break. Similarly, an earlier study carried out in the USA found that during the summer break from school, children’s weight increased more quickly than it did during term time, demonstrating that children are at risk from rapid weight gain during the school holidays (16). The authors proposed that the school environment has a positive influence on children’s weight status and that summer interventions are needed to attenuate children’s weight gain outside of the school term.

A lack of engagement in structured activity during the school holidays is also thought to have a detrimental impact on children’s learning and attainment, especially during the extended summer break from school. Summer learning loss refers to the tendency for children to fall behind academically when they are not engaged in learning activities during school holidays (17), with mathematics and spelling performance reported to be particularly susceptible to decline (18).

In order to address the need for food and activity provision during the school holidays, a number of different schemes have been implemented across the UK to provide families, particularly those on low incomes, with meals and opportunities to engage in enrichment activities during the school holidays^2^. However, at the current time, very little is known about holiday provision in the UK and there is a general lack of academic research reporting on the efficacy of UK-based holiday clubs. Moreover, existing research in this area has adopted a limited focus by investigating holiday breakfast clubs based only in community venues, such as church halls and food banks (11).

The current study set out to address some of the limitations of prior research, in terms of the limited focus on community venues in the North West of England and Northern Ireland, by investigating holiday clubs based in school and community settings across the South of England and Wales, UK. Drawing on the views of holiday club staff, the current study aimed to answer three research questions: (1) Why is there a need for holiday clubs? (2) What are the benefits of holiday club participation? and (3) What factors need to be considered in the development of holiday club provision?

## Materials and Methods

### Approach

A semi-structured interview approach was adopted for the purpose of the current study. This method was deemed appropriate as it allowed participants to talk openly about sensitive topics, such as food insecurity and to freely express their views about the running of their holiday clubs. Interviews were carried out via the telephone so that staff could participate at a time that was appropriate to them without interfering with their day to day tasks within the holiday club setting.

### Participants

Fourteen staff members (1 male and 13 females) from six holiday clubs were purposively sampled to participate in the current study. Purposive sampling refers to the method of selecting participants based on a particular characteristic that is relevant to the investigation (19). Staff involved in the current study were purposively sampled as they were all involved in the day to day running of a UK-based holiday club during summer 2015. While the purposive selection of participants might introduce some bias, it has been argued that a purposive sample is an essential component in the collection of valuable qualitative data (19).

The holiday clubs took place in school and community-based premises across the South of England and Wales for approximately 4–6 weeks of the school summer break. A summary of the features of each holiday club is presented in Table 1 with each individual club referred to by number rather than name in order to uphold anonymity.

**Table 1:** **Characteristics of participating holiday clubs**.

Club	Number of staff interviewed	Location	Setting	Led by	Year of operation	Availability	Provision	Family involvement
1	1	Wales	Primary school	School staff	First year	9:30–1:00; 3 days per week	Breakfast, lunch, and activities (crafts, sports, and nutrition)	Family lunch offered 1 day per week
2	3	Wales	Primary school	Housing association staff and sports coaches	Tenth year but first year of food provision	9:30–1:00; 3 days per week	Breakfast, lunch, and sports activities	Family lunch offered 1 day per week
3	1	Wales	Primary school	School staff	First year	9:30–1:00; 3 days per week	Breakfast, lunch, and activities (crafts, sports, and nutrition)	Family lunch offered 1 day per week
4	4	Wales	Primary school	School staff	First year	9:30–1:00; 3 days per week	Breakfast, lunch, and activities (crafts, sports, and nutrition)	Family lunch offered 1 day per week
5	2	Southern England	Town hall	Volunteers	First year	12:00–1:30; 3 days per week	Lunch and activities (crafts and board games)	Families welcome to attend daily
6	3	Southern England	Church hall	Volunteers	Third year	11:30–1:15; 3 days per week	Lunch and activities (crafts and board games)	Families welcome to attend daily

### Materials

A semi-structured interview schedule was developed for the purpose of the current investigation, consisting of a series of open questions that invited participants to talk freely about various aspects of their holiday clubs. A copy of the interview schedule is included in Table 2.

**Table 2:** **Interview schedule**.

Can you tell me a little bit about your holiday club and what it offers children and families day to day?
What are your responsibilities in the holiday club?
What made you decide to become involved in the holiday club?
How were children selected to attend your holiday club?
What do you think the reasons are for children attending the holiday club?
Overall, what do you think children and parents gain from attending the holiday club?
What do staff gain from their involvement in the holiday club?
Are there any drawbacks to the holiday club?
Could you tell me a little bit about what you serve?
How do you think these foods and drinks are different to what children usually have at home?
How do you think the foods and drinks at holiday club might have made a difference to families during the summer break?
If holiday clubs were to run again, would you like to be involved? Why?
Do you think (your school/community setting) is a good venue for the holiday club? Why?
Is there anything that you didn’t like about this year’s holiday club?
What could be done to improve holiday club in the future?
Is there anything else that you’d like to mention about holiday clubs that we haven’t covered already?

### Procedure

Following ethical approval from Northumbria University Ethics Board (July, 2015), information about the research project was posted out to each holiday club. Completed consent forms were then collected from each holiday club by a member of the research team during August 2015 and those who provided written consent to participate were subsequently contacted and interviewed via telephone in September 2015. The interviews, which were carried out by a research assistant from Northumbria University, followed a semi-structured format to allow the interviewer to gain further insight and clarification on points of interest where necessary. All interviews were audio recorded and transcribed verbatim for subsequent thematic analysis. On average, the interviews lasted 18 min. Transcription was carried out by the interviewer and the accuracy of the transcripts was subsequently checked against the recordings by a second researcher. Thematic analysis was carried out by the second researcher and second coded by the interviewer as detailed under the following section.

### Analysis

It has been proposed that qualitative interviews should continue until a saturation point is reached. Saturation refers to the stage at which no new themes are identifiable within the data and the inclusion of further interviews does not serve to add new themes to the data set (20, 21). In the current study, the analytical process was carried out in parallel with interviews to allow the research team to determine when a saturation of themes was reached.

Data were coded and analyzed in accordance with published guidelines on the process of thematic analysis (22). An inductive approach to thematic analysis was adopted as there are currently no published theoretical frameworks available relating to holiday provision that could have been used for the purpose of data analysis in the current investigation. Each individual interview recording was listened to in its entirety before being orthographically transcribed. Transcripts were read multiple times and key quotes thought to address each individual research question were highlighted. These quotes were then given initial codes relating to their content and grouped based on similarity of content. Main themes were developed from these topic groups, and appropriate theme headings were generated to represent the content of each theme. Subsequently, a sample of around 10% of the coded transcripts were analyzed by a second coder to confirm the accuracy of the analysis. The level of agreement between the first and second coder was found to be substantial (Cohen’s kappa = 0.727) according to Landis and Koch’s (23) Kappa statistic interpretation criteria.

### Findings

Staff reflected on their knowledge of their local area and experiences of working in holiday clubs to share their views on the need for holiday club provision; the benefits of holiday club participation for children and adults; and potential areas for further development of holiday clubs. A summary of the themes that were identified across staff interviews is subsequently presented with themes, initial codes, and example quotes presented in Table 3.

**Table 3:** **Summary of themes, codes, and example quotes**.

Research question	Theme	Codes	Example quotes
Why is there a need for holiday clubs?	Holiday hardship	Lack of food at home Limited range of food at home Poor food choices at home Families struggle during the holidays All levels of society are struggling	*If somebody’s got 3 or 4 children and suddenly they’ve got to provide a meal in the middle of the day as well, you know, that can knock their budgeting way out the window yeah some of them are sort of living on the edge as it were* (Club 6)
	Evidence of demand	Attendance increased Valued resource Children kept returning Families are referred	*It was well worth you know putting on the club and it was you know well accepted by the parents as well they thought it was a great idea and quite a few have come back and asked if we are running it again next year already* (Club 1)
	Inactivity and isolation	Children lack attention at home/get bored/are inactive Families lack things to do Parents become isolated	*Some of them need it because they haven’t got that good at home life so it’s great for them to just get- for two hours and be- you know, get a bit of attention and enjoy themselves really* (Club 4)
What are the benefits of holiday club participation?	Access to food	Access to a variety of foods Access to healthier foods than at home Access to new foods Food for wider family Food provision valued	*I think for some of the children because they were entitled to free school meals I think it was- well I think the parents quite liked that they were able to have breakfast and dinner* (Club 4)
	Access to activities	Enjoyable Accessible activities Something to do Reduced boredom Children more active Reduced likelihood of problem behavior	*An opportunity for children to take part in various sports and activities. Some of these children may not have opportunities to visit the leisure centre in the area* (Club 2)
	Social opportunities	Age groups mixing Bringing cultures and families together Social time for children, parents, and staff Breaks down barriers between home and school Staff get to know children and families better	*They get to interact with other kids irrespective of age, race yeah everyone’s just one big family* (Club 6)
	Familial support	Eases pressure on parents Gives parents a break Safe environment for children Peace of mind for parents Saved parents money – food, activities, child care Supportive environment	*I think it’s saved some of the parents- it didn’t cost them as much during the summer, they didn’t have to pay out so much to go and do things, to go and buy you know their lunch* (Club 2)
	Informal learning experience	Children learn new skills Cooking tips for parents Children did not realize they were learning Informative for parents Knowledge passed on at home	*They didn’t realize they were doing nutrition so we bought boxes of cereals and brought boxes from home so they could cut out and you know make a collage* (Club 1)
	Positive experience for staff	Enjoyable for staff Satisfaction of helping others Valuable experience for staff Extra income for staff	*I thoroughly enjoyed being part of it* (Club 4)
What factors need to be considered in the development of holiday club provision?	Widening participation	Limited uptake Difficult to engage some parents Difficult to target- stigma Open to wider community Combine provision across schools	*I think we still got a way to go to really encourage those people who need to come I think some people are too proud to say I need this and I would really benefit from this* (Club 5)
	Prior planning	Disorganized to start Lack of prior planning Duration needs consideration More planning time needed Risk of double breakfast Include more activities Involve children in planning Experience could feed into future provision	*It was a little disorganized to start with I think like there was quite a few organizations involved so some of the communication between what we were meant to be doing how things were going to be done who was coming in to do what were a little bit kind of confused at times* (Club 3)
	Staffing considerations	Additional staff needed Good to draw on expertise/community support Big time commitment Missing staff can be problematic Inconsistencies in staff pay	*Sometimes we do run short of volunteers so any more we do get would be nice* (Club 6)
	Holiday club setting	Need to consider amenities Kitchen access is advantageous School is a good venue- familiar setting, access to resources, wasted when closed during holidays Church venue was not limiting Outdoor space is useful	*I think it was excellent to be honest because everything was there it- it seems a waste of a building to shut it for six weeks when you could use it* (Club 1)

### Why Is There a Need for Holiday Clubs?

Three themes relating to staff views on the need for holiday clubs were established through interviews with holiday club staff: *Holiday Hardship; Inactivity and Isolation; and Evidence of Demand*.

Staff believed that holiday clubs were a worthwhile provision to support struggling families during holidays from school and believed that they should be made available in future. They were aware of families from various income levels being heavily reliant on school meals during term time and expressed concern that a lack of school meal provision during the school holidays could lead to children skipping meals or being provided with a limited range of foods, which are low in nutritional value. Furthermore, financial hardship was thought to limit family engagement in activities during the school holidays, which led to children and parents becoming quite sedentary and isolated within their communities.

Staff reflections on their experiences of holiday clubs suggested that uptake was relatively low compared to their expectations. However, staff reported that attendance increased across the duration of the holidays and those families who attended did so consistently, thus evidencing a demand for the provision.

### What are the Benefits of Holiday Club Participation?

Six key themes encompassed the benefits of holiday clubs discussed by staff: *Access to Food; Access to Activities; Social Opportunities; Familial Support; Informal Learning Experience*; *and Positive Experience for Staff*.

In addition to providing a reliable and essential source of food for some children and their families, staff felt that the food provided at holiday clubs gave children opportunities to try new and more nutritious foods than they would have access to at home. Staff believed that the children liked trying new foods and felt that these new food experiences could have a positive influence on children’s dietary habits at home.

In addition to food, holiday clubs offered an array of enjoyable activities for children to partake in each day. The activities on offer were valued as they were deemed to be more accessible than other activities on offer in the local area; they were believed to alleviate boredom and reduce the likelihood that children would engage in anti-social behavior. Moreover, children and adults learnt new skills and knowledge through the holiday club activities, particularly in relation to food, nutrition, and sports skills. The informal nature of these activities meant that children often did not realize that they were engaging in learning activities.

The provision of food and activities provided a platform for children and adults to interact informally, creating a strong social element to the holiday clubs. Children, parents, and staff were able to spend time and build relationships with others that they would be unlikely to encounter at any other time. Moreover, staff felt that holiday clubs helped to break down perceived barriers to parents engaging with school and community interventions.

Holiday clubs were also viewed as valuable sources of support, providing an environment where parents and children could go for various kinds of help, beyond the provision of food. By offering an accessible package of food, activities, and child care, holiday clubs were suggested to alleviate financial constraints on parents during the school holidays, while providing parents with peace of mind that their children were in a safe and fun environment.

Finally, the staff involved in the holiday clubs found their experiences to be very rewarding. They gained satisfaction from helping others in their communities, gained professional development experience, and some benefited from earning extra income during the school holidays.

### What Factors Need to be Considered in the Development of Holiday Club Provision?

On the whole, staff were very positive about holiday club provision and, as evidenced through their views on the benefits of holiday club participation, staff felt that holiday clubs had a lot to offer to children, parents, and staff. However, reflections on their own experiences led staff to suggest some minor areas for consideration should holiday clubs be implemented longer term. Potential areas for development discussed by staff were summarized across four themes: *Staffing Considerations; Prior Planning; Widening Participation; and Holiday Club Setting*.

First, holiday clubs tended to bring together a number of organizations, including local authority representatives, school staff, and volunteers. Though staff recognized value in being able to draw on the expertise of various agencies, staff suggested that there needs to be more open communication between organizations to ensure that everyone is aware of their individual roles and responsibilities, and to make sure that nothing is overlooked. Moreover, some staff believed that taking on the responsibility of working in a holiday club was a big commitment and clubs could benefit from additional staffing. This was particularly the case for those clubs that relied on volunteers to work in the clubs day to day as they sometimes faced difficulties with having fewer staff than anticipated. Though when staff were paid for their time, there were issues with staff being paid different hourly rates for doing the same job as holiday club rates of pay were based on their term time rates of pay. This led to concerns that such issues with pay might deter people from becoming involved in the delivery of holiday clubs in future.

Second, some staff felt that they had very little planning time prior to the commencement of the holiday clubs and believed that this would need to be addressed in future. Staff hoped to have more autonomy in terms of the activities they offered in future but, for those clubs in their first year of operation, they believed that they now had a bank of resources and experiences that they could draw upon in future.

Furthermore, in terms of planning, it emerged that there were mixed views on the appropriate duration for the holiday clubs to run. Some staff felt that only offering provision for part of the week was good as it allowed children and staff some time away, while others suggested that it would have been useful to run for more days throughout the holidays or for a longer time period each day. Interestingly, in those clubs where breakfast was served, the start time of the clubs was thought to have an impact on children’s perceptions of the breakfast meal, which consequently impacted how much food they consumed in the mornings as some children had breakfast at home then went on to have more breakfast when they arrived at the holiday club.

A further consideration for future holiday provision related to attendance rates. Although staff saw an obvious need for holiday clubs, the number of people attending the clubs overall was relatively low, which prompted staff to propose that more work would be necessary in future to ensure that those families who could benefit most from holiday provision are reached. However, it was suggested that directly targeting children and families to attend reduced opportunities for advertising and could potentially lead to attendance being stigmatized.

Finally, staff across school and community settings spoke favorably about the venues that their holiday clubs took place in. Schools were thought to be particularly well placed to host holiday clubs given children’s familiarity with them and the abundance of resources and outdoor space that they offered; though the use of community venues was not thought to limit families’ holiday club enjoyment or access. Clubs based in community venues were more limited than schools in terms of outdoor space. However, all the holiday club venues had ample kitchen space and amenities to be able to offer cooked meals and this was something that staff recognized as important.

## Discussion

The aim of the current study was to investigate the need for holiday club provision, the benefits of holiday club participation, and potential areas for future development from the perspective of holiday club staff. The findings of the investigation showed that staff believe there is a need for holiday club provision to help to relieve financial strain on families and to encourage them to remain active and engaged within their communities throughout the school holidays. Staff believed that holiday club participation conferred a range of benefits for families by providing access to meals and activities and offering social, learning, and support opportunities. Additionally, staff felt that they had benefited from their involvement in holiday clubs. They gained enjoyment and satisfaction from helping people within their communities and some benefited from additional income. Staff hoped to see holiday clubs made available in the future but were keen to see more careful planning and deployment of staff in future provision.

The views of staff presented in the current study lend support to practitioners and policymakers who have argued that there is a need for accessible food and activity provision during the school holidays for children and families in the UK (24). There is ample evidence demonstrating the need for a nutritious diet and regular physical activity throughout childhood (4–6, 14). However, evidence presented in the current study suggested that during the school holidays, many children engage in poor dietary habits and excessive sedentary behavior, which may potentially have a detrimental impact on longer term health outcomes. In line with previous research (16), which has shown that children’s weight increases at a faster rate during the school holidays than in term time, the current findings provide support for the implementation of school holiday interventions to promote positive health behaviors.

Through food and activity provision, holiday clubs in the current study were also thought to be able to provide a mechanism of support for families. In addition to relieving financial pressures, holiday clubs offered children and parents some respite and somewhere to socialize regularly with people from their local area. This is important as previous research has shown that children and parents are at risk of becoming isolated during the school holidays and generally receive a lack of support from their families and communities (12). Such isolation and a lack of social support can have unfavorable outcomes for health and wellbeing (25, 26). Therefore, by reducing the risk of isolation, holiday clubs have the potential to support the health and wellbeing of children and parents in ways that extend beyond food and activity provision alone.

Additionally, by supporting families through the provision of accessible childcare, holiday clubs were suggested to help parents to remain in employment and keep children engaged in activities, thus ensuring maintenance of a consistent routine, income stability for parents and less boredom for children. Given that The Trussell Trust have reported income instability as a key reason for people having to rely on food banks (see text footnote 1), supporting parents to retain employment during the school holidays could lead to a reduction in the high prevalence of families accessing food aid outside of school term time. Moreover, the prevalence of anti-social behavior tends to increase during the school holidays (27) and some anecdotal evidence suggests that the prevalence of children engaging in unfavorable, anti-social behaviors decreases when they attend activities-based school holiday programs (28). This may occur because holiday clubs help to increase positive peer associations and, at the same time, decreases the probability that youth will engage in drug use (29). Furthermore, holiday clubs may contribute to the overall number of community youth organizations in operation, which is found to be inversely correlated with lower levels of neighborhood youth violence (30). Therefore, if holiday clubs help to reduce engagement in anti-social behavior, then investment in holiday clubs potentially has wider societal benefits.

A final key benefit of holiday clubs outlined by staff in the current study related to the learning opportunities that children and families were afforded. In some cases, learning took place through sessions designed to impart knowledge and skills, such as nutrition lessons and sports coaching. However, some staff felt that families were learning and being inspired to try new things simply by participating in holiday clubs. Such direct and indirect learning opportunities are particularly important for children, given concerns that during the summer break from school, children are at risk from summer learning loss (16, 17). Staff in the current study highlighted that children enjoyed taking part in the activities that were available to them; thus, holiday clubs could provide a useful outlet for summer learning, potentially reducing the prevalence of learning loss when children return to school in the new term. However, it is important to note that staff were keen to ensure that holiday club activities were fun and engaging for children and did not become too much like typical school day activities.

The current findings offer a timely contribution to the research literature in this area given recent calls by UK policymakers and practitioners for more research to be carried out to investigate the issue of holiday hunger and the impact of holiday provision (24). The views of staff in the current investigation highlight the value of holiday clubs across communities of varying levels of deprivation and show the potential for school and community-based clubs to support families in ways that extend beyond the alleviation of hunger. Nevertheless, it is important to acknowledge that the current paper reports on the views of a small, purposive sample of holiday club staff; thus, these views might not be representative of a much more diverse sample of staff spanning other geographical areas. Though, it is noteworthy that the need for holiday food provision and the various benefits of holiday provision for parents, children, and staff outlined by Defeyter et al. (11) are supported by the themes identified in the current paper. A promising direction for work in this area would, therefore, be to quantitatively measure the health, social and educational outcomes relating to holiday clubs that have emerged through qualitative work to date. Furthermore, future work would also benefit from a health economics evaluation in order to fully determine the value of investment in holiday provision.

To conclude, the current investigation provides a useful contribution to the limited academic work in this area and provides a point of reference for policymakers, stakeholders, and academics with an interest in holiday clubs.

## Author Contributions

PG contributed to study design, recruitment, analysis, and manuscript preparation. EC contributed to data collection, analysis, and manuscript preparation. PS contributed to study design and manuscript preparation. ML contributed to study design and manuscript preparation. KP contributed to study design, recruitment, and manuscript preparation. ES contributed to study design, recruitment, and manuscript preparation. MD contributed to study design, recruitment, analysis, and manuscript preparation.

## Conflict of Interest Statement

The authors declare that the research was conducted in the absence of any commercial or financial relationships that could be construed as a potential conflict of interest.

## Footnotes

^1^https://www.trusselltrust.org/news-and-blog/latest-stats/

^2^http://www.makelunch.org.uk
